# Improving cytocompatibility of CdTe quantum dots by Schiff-base-coordinated lanthanides surface doping

**DOI:** 10.1186/s12951-018-0369-7

**Published:** 2018-04-19

**Authors:** Hana Buchtelova, Vladislav Strmiska, Zuzana Skubalova, Simona Dostalova, Petr Michalek, Sona Krizkova, David Hynek, Lukas Kalina, Lukas Richtera, Amitava Moulick, Vojtech Adam, Zbynek Heger

**Affiliations:** 1 Department of Chemistry and Biochemistry, Mendel University in Brno, Zemedelska 1, 613 00 Brno, Czech Republic; 2 Central European Institute of Technology, Brno University of Technology, Purkynova 123, 612 00 Brno, Czech Republic; 3 Materials Research Centre, Faculty of Chemistry, Brno University of Technology, Purkynova 118, 612 00 Brno, Czech Republic

**Keywords:** Cellular labeling, Cytotoxicity, Inorganic fluorophore, Nanocrystal, Surface dopant

## Abstract

**Background:**

Suitable fluorophores are the core of fluorescence imaging. Among the most exciting, yet controversial, labels are quantum dots (QDs) with their unique optical and chemical properties, but also considerable toxicity. This hinders QDs applicability in living systems. Surface chemistry has a profound impact on biological behavior of QDs. This study describes a two-step synthesis of QDs formed by CdTe core doped with Schiff base ligand for lanthanides [Ln (Yb^3+^, Tb^3+^ and Gd^3+^)] as novel cytocompatible fluorophores.

**Results:**

Microwave-assisted synthesis resulted in water-soluble nanocrystals with high colloidal and fluorescence stability with quantum yields of 40.9–58.0%. Despite induction of endocytosis and cytoplasm accumulation of Yb- and TbQDs, surface doping resulted in significant enhancement in cytocompatibility when compared to the un-doped CdTe QDs. Furthermore, only negligible antimigratory properties without triggering formation of reactive oxygen species were found, particularly for TbQDs. Ln-doped QDs did not cause observable hemolysis, adsorbed only a low degree of plasma proteins onto their surface and did not possess significant genotoxicity. To validate the applicability of Ln-doped QDs for in vitro visualization of receptor status of living cells, we performed a site-directed conjugation of antibodies towards immuno-labeling of clinically relevant target—human norepinephrine transporter (hNET), over-expressed in neuroendocrine tumors like neuroblastoma. Immuno-performance of modified TbQDs was successfully tested in distinct types of cells varying in hNET expression and also in neuroblastoma cells with hNET expression up-regulated by vorinostat.

**Conclusion:**

For the first time we show that Ln-doping of CdTe QDs can significantly alleviate their cytotoxic effects. The obtained results imply great potential of Ln-doped QDs as cytocompatible and stable fluorophores for various bio-labeling applications.

## Background

Quantum dots (QDs) are semiconductor nanocrystals (~ 2 to 10 nm) with unique optical and electrical properties [1]. Due to these properties, QDs have undisputable potential to revolutionize biological imaging and to become a new class of fluorescent probes [2]. Emission spectra of QDs are narrow, symmetrical, and tunable according to their size and material composition [3]. In addition, QDs possess considerable photostability and chemical stability compared with organic dyes and fluorescent proteins [4–6]. These properties permit the efficient excitation of multiple-color QDs with a single light source and predispose QDs for the long-term labeling and monitoring of live cells, which is a crucial technique in cell biology [7].

One aspect of QDs that has been largely discussed is the QD-associated cytotoxicity in comparison to that of traditional organic cell-labeling fluorescent probes that are commonly applied for live cell labeling and imaging [8]. Among II–VI semiconductor compounds, CdTe has attracted much interest due to their high quantum yield (QY) and a possibility of their single-pot synthesis in aqueous phase [9, 10]. In the early 2000s, scientists became aware that the CdTe QDs exert toxic effects including chromatin condensation or membrane blebbing, both features associated with apoptosis [7, 11]. One of the facets contributing to CdTe QDs toxicity is the contamination of QDs-containing solution with soluble Cd^2+^ or Cd^2+^ released from QDs. Moreover, physico-chemical attributes, such as size, charge or surface capping can influence toxicity of QDs [12]. Therefore, to achieve cyto- and biocompatibility, attention has been concentrated towards carbon-based QDs [13], or surface modifications of metal-based QDs [14].

In this study, Ln^3+^ (Yb^3+^, Tb^3+^ and Gd^3+^)-Schiff base-doped CdTe QDs were synthesized and characterized. The preparation of Ln^3+^-doped wide band gap QDs has actually been explored for many years [15, 16]. Ln-doped QDs are advantageous over other QDs due the sharp emission signal from Ln^3+^ having a unique spectroscopic signature. The long Ln fluorescence lifetime also helps to distinguish the signal from the background autofluorescence of biological media [17]. Although no apparent cytotoxicity has been described for the Ln-doped up-conversion nanoparticles (NaYF_4_, CaF_2_ or SrF_2_ using Yb^3+^, Er^3+^ or Tm^3+^ as dopants) [18–20], there is still a lack of data regarding cytotoxicity and biocompatibility of Ln-doped QDs.

Hence, the main aim of this study was to generate and analyze a homogenous set of experimental data describing the link between cellular responses and exposure to Ln-doped QDs. We focused on evaluation of viability, endocytosis, antimigratory properties, induction of formation of reactive oxygen species (ROS) and stimulatory/inhibitory effect of Ln-doped QDs on the expression of proteins involved in fundamental biological processes, including apoptosis, cell cycle, signaling or metal homeostasis. We also investigated in vitro biocompatibility of Ln-doped QDs in terms of hemocompatibility, genotoxicity, and formation of protein corona.

Finally, to validate the applicability of Ln-doped QDs for the in vitro visualization of receptor status, site-directed conjugation of antibodies onto Ln-doped QDs through peptide linker was optimized. We focused on immuno-labeling of clinically relevant target—human norepinephrine transporter (hNET), which is over-expressed in neuroendocrine tumors like neuroblastoma, and which is targeted by one of the most widely used theranostic agent metaiodobenzylguanidine (mIBG) [21].

Overall, Ln-doping results in a significant increase of cytocompatibility of CdTe QDs and provides exceptional cytocompatible and stable inorganic fluorophores for in vitro immuno-labeling.

## Results

### Physico-chemical attributes of Ln-doped-QDs

**Fig. 1:**
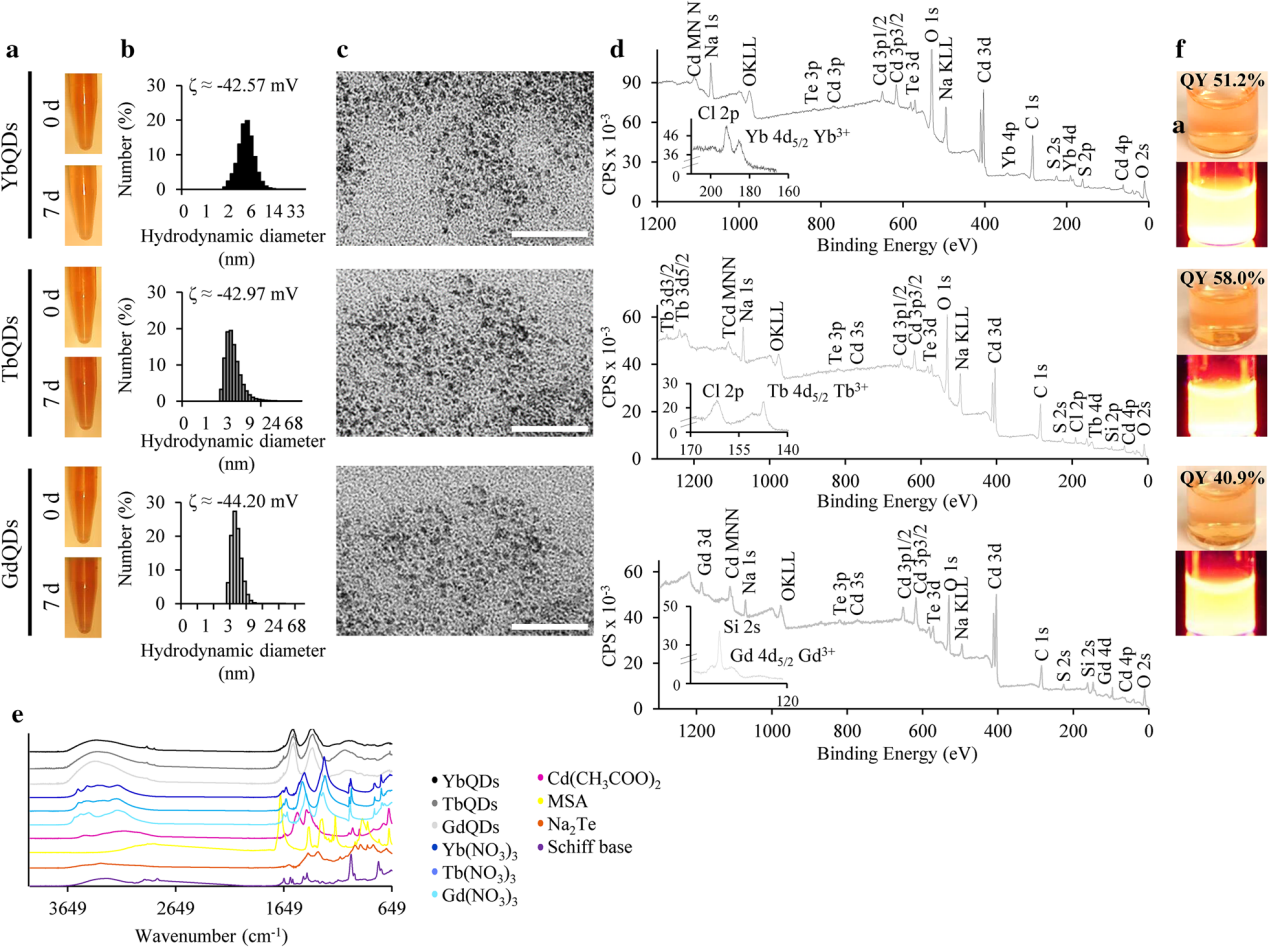
Characterization of Ln-doped QDs. **a** Photodocumentation of a colloidal stability of QDs showing their stability without sedimentation at start-point (0 h) and upon 7 days of storage at 25 °C in RS. **b** Corresponding size distribution histograms analyzed by quasielastic DLS. Inserted are ζ-potential values analyzed in RS 100-fold diluted with Milli-Q water (pH 7.4) by Doppler microelectrophoresis. **c** TEM micrographs with the length of scale bar 50 nm. **d** XPS survey spectra and **e** FT-IR spectra of Ln-doped QDs and individual components used for synthesis. **f** Photographs of Ln-doped QDs in ambient light and after exposure to UV transillumination (λ_exc_ = 312 nm). Inserted are QY values determined using rhodamine 6G as a reference

After microwave-assisted doping of CdTe QDs with Schiff base bearing selected Ln (Yb, Tb and Gd), we analyzed their physico-chemical attributes. Figure 1a shows that all three types of Ln-doped QDs were found to disperse readily in Ringer’s solution (RS) and remained stable in dispersion for more than 7 days. This was confirmed by analyzing the ζ-potentials (ranging from − 42.57 to − 44.20 mV, inserted in Fig. 1b), which are the key indicators of stability of colloidal dispersion. Dynamic light scattering (DLS) histograms depicted in Fig. 1b demonstrated slight differences in size distributions among individual Ln-doped QDs. The largest hydrodynamic diameter (HDD) was identified for YbQDs (the highest distribution at ~ 5.4 nm), followed by GdQDs (~ 4.1 nm) and TbQDs (~ 3.9 nm), which were a bit larger than that of an un-doped CdTe QDs (~ 3.1 nm). Transmission electron microscopy (TEM) micrographs confirmed relatively uniform size distribution of Ln-doped QDs without obvious aggregation (Fig. 1c). In addition, X-ray photoelectron spectroscopy (XPS) showed elemental composition of Ln-doped QDs and revealed that the binding energies of Ln-Schiff base (Yb4d_5/2_ = 185.3 eV; Tb4d_5/2_ = 147.3 eV; Gd4d_5/2_ = 143.2 eV) corresponded to the Ln^3+^ oxidation state (Fig. 1d). Similarly, the presence of Schiff base on Ln-doped QDs was confirmed using Fourier transform infrared spectroscopy (FT-IR, Fig. 1e). The essential parameter to evaluate the light emission properties of fluorophores is QY, quantifying the conversion efficiency of adsorbed to emitted photons. QY of Ln-doped QDs ranged between 40.9% for GdQDs to 58.0% for TbQDs (Fig. 1f). Under UV transillumination, all Ln-doped QDs displayed a bright yellow-to-orange color.

### Optical stability of Ln-doped QDs in physiological environments

**Fig. 2:**
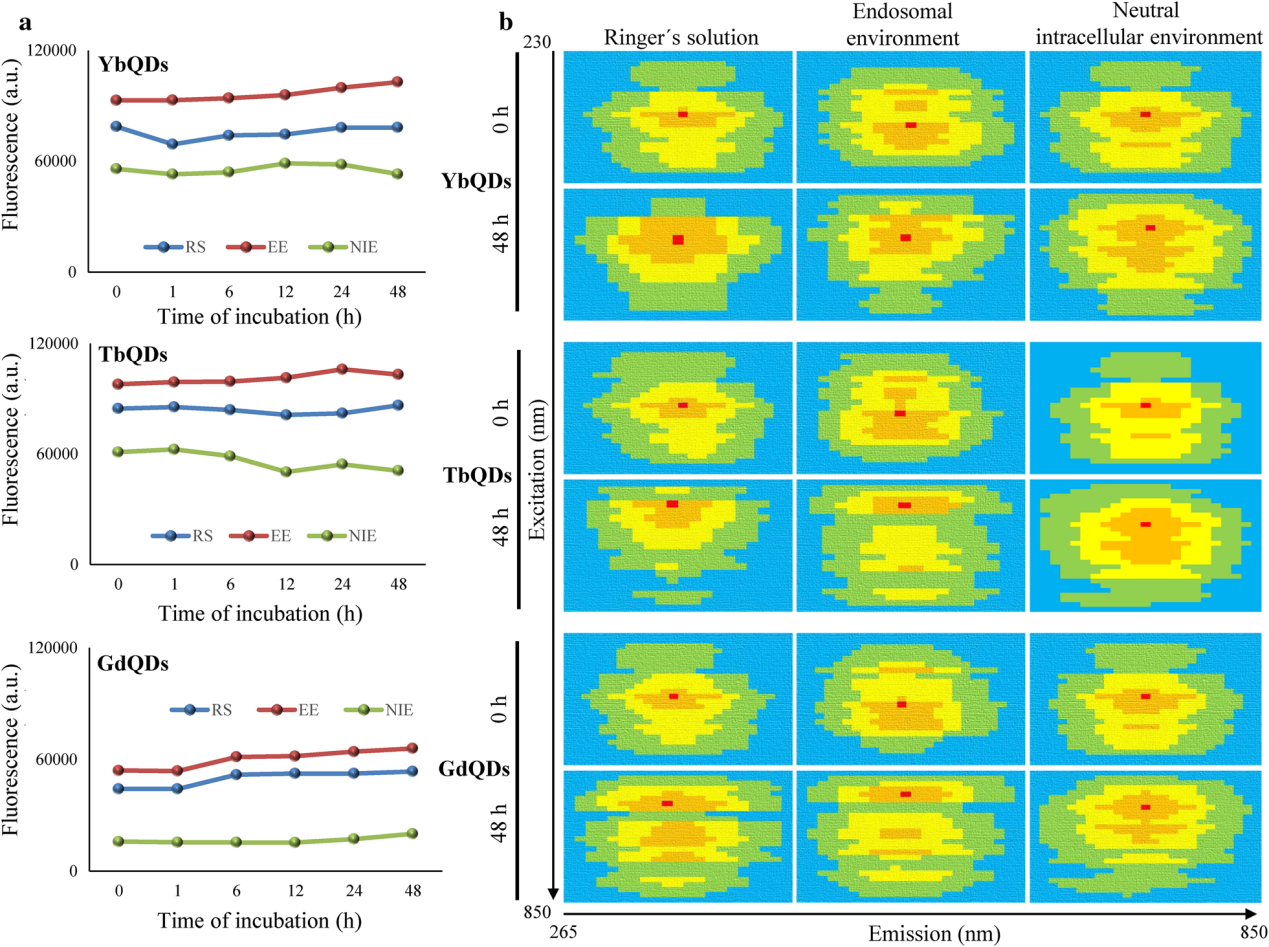
**a** Fluorescence stability of Ln-doped QDs incubated (up to 48 h) in solutions mimicking distinct physiological environments. *RS* Ringer’s solution, *EE* endosomal environment, *NIE* neutral intracellular environment. **b** 2D fluorescence emission–excitation spectral maps of Ln-doped QDs obtained upon incubation (48 h) in various solutions mimicking physiological conditions (plasma, endosomes and cytoplasm). Spectra illustrate environment-dependent optima for excitation and emission. Red spots highlight maximum performance of fluorescence, blue spots—no or negligible fluorescence recorded

To extend the application of QDs to bio-labeling applications, fluorescence stability in physiological environment is a key prerequisite. Therefore, we performed a systematic investigation of fluorescence of Ln-doped QDs in solutions mimicking physiological environments. Noteworthy, Fig. 2a demonstrates that upon 48 h incubation, no significant quenching occurred in any of tested Ln-doped QDs. Interestingly, the highest emission was yielded in solution mimicking endosomes (EE), followed by RS and neutral intracellular fluid (NIE). The marked decrease in emission upon incubation in NIE was accompanied by the increase in HDD of all tested Ln-doped QDs as summarized in Table 1. In agreement with the lowest QY value, GdQDs demonstrated also the lowest emission yield, irrespective to incubation environment.

**Table 1:** Time-course development of HDD (nm) of Ln-doped QDs incubated in RS, EE and NIE

	YbQDs			TbQDs			GdQDs		
	*RS*	*EE*	*NIE*	*RS*	*EE*	*NIE*	*RS*	*EE*	*NIE*
0 h	5.4	5.5	5.4	4.1	4.2	4.0	3.2	3.0	3.1
1 h	5.6	5.2	5.9	4.3	4.7	4.8	3.4	3.5	3.8
6 h	5.4	5.9	6.2	4.4	4.3	5.3	3.6	3.5	4.2
12 h	5.8	5.5	6.9	4.2	4.5	5.5	3.7	3.4	4.1
24 h	5.6	5.4	7.2	4.4	4.2	6.2	3.5	3.6	5.2
48 h	5.9	5.6	8.0	5.1	4.6	6.0	3.6	3.7	6.9

The results are expressed as mean HDD (nm) of three independent analyses

Global views on 2D fluorescence emission–excitation spectral maps are shown in Fig. 2b. It clearly follows from the maps that incubation had only negligible effects on emission maxima. Contrary to that, excitation maxima were shifted to lower wavelengths. This was most significant for incubation in RS and EE. These properties have to be characterized and taken into account prior to any bio-labeling applications.

### Ln-doping pronouncedly increased cytocompatibility of CdTe QDs

**Fig. 3:**
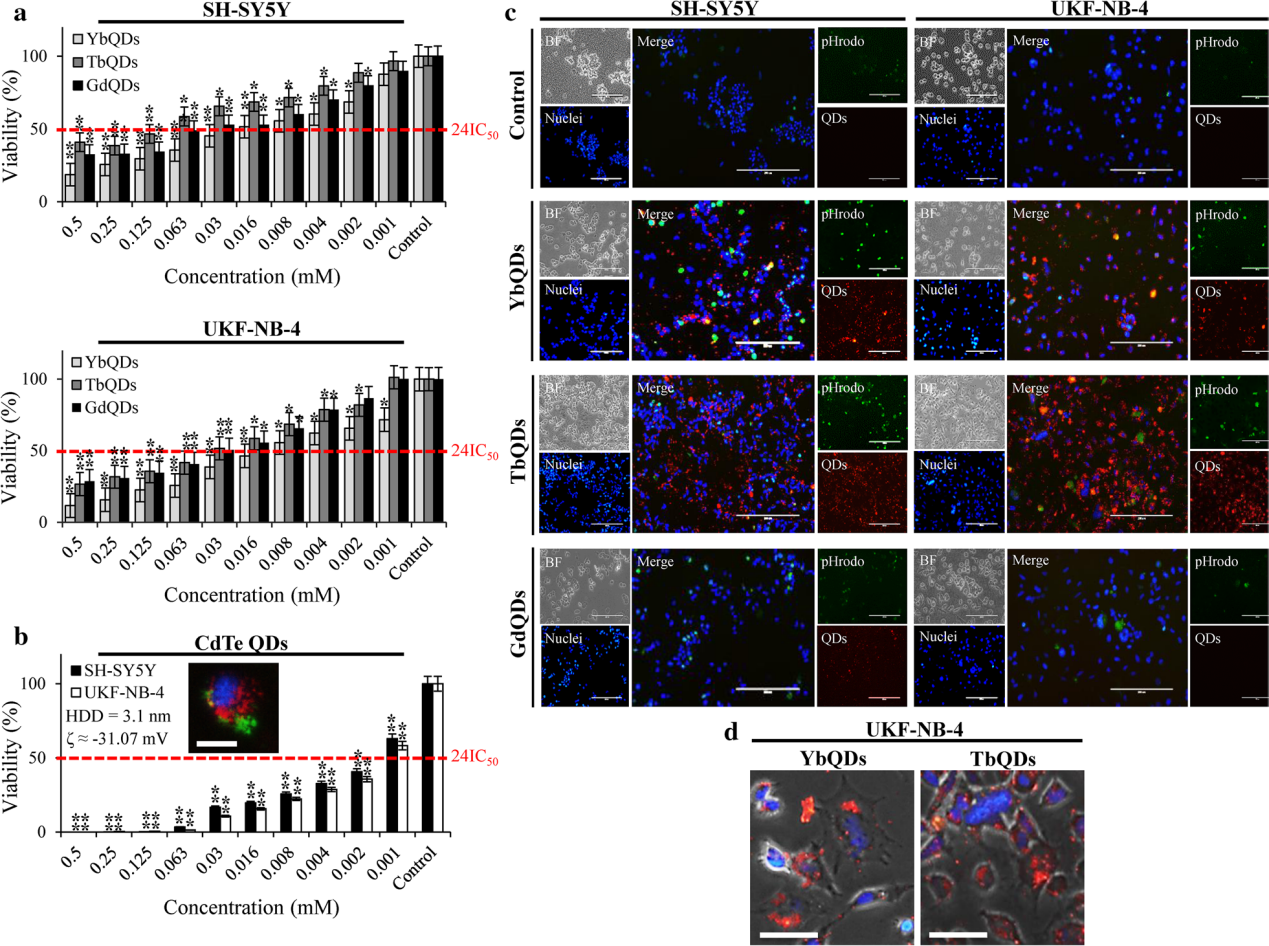
**a** Cytotoxicity assays showing viability of tested neuroblastoma cells after incubation with Ln-doped QDs (0.001–1 mM, 24 h). **b** MTT assay of green CdTe QDs illustrating their pronounced cytotoxicity in comparison to Ln-doped QDs. Inserted are basic characteristics of CdTe and image depicting their endocytosis (red, 10,000 MW pHrodo Red) in UKF-NB-4 cells. Scale bar, 20 μm. Red line indicates approximate 24IC_50_ values. The values are expressed as the mean of six independent replicates (*n* = 6). Vertical bars indicate standard error. *p < 0.05, **p < 0.01 related to control non-treated cells. **c** Living cells fluorescence microscopy showing Ln-doped QDs-induced endocytosis (10,000 MW pHrodo Green). Scale bars, 200 μm. *BF* bright field image. **d** Cytoplasmic accumulation of YbQDs and TbQDs in UKF-NB-4 cells. Scale bars, 40 μm. In all microscopic analyses, nuclei were counterstained with Hoechst 33342. Prior to microscopy, cells were incubated with 2 μM QDs for 6 h

Next, we focused on cytotoxic screenings of Ln-doped QDs using two types of neuroblastoma cells (SH-SY5Y and UKF-NB-4). Figure 3a illustrates that in both cell lines, Ln-doping significantly reduced cytotoxicity of un-doped CdTe QDs (Fig. 3b). The highest cytotoxic effect was caused by YbQDs (24 h IC_50_ ~ 0.022 mM for SH-SY5Y and 0.011 mM for UKF-NB-4), followed by GdQDs (24 h IC_50_ ~ 0.026 mM for SH-SY5Y and 0.033 mM for UKF-NB-4) and TbQDs (24 h IC_50_ ~ 0.139 mM for SH-SY5Y and 0.038 mM for UKF-NB-4). To shed light on the interaction between Ln-doped QDs and cells, we further studied accumulation of Ln-doped QDs in intracellular space of neuroblastoma cells. Figure 3c shows that all tested Ln-doped QDs caused marked induction of endocytosis, which has been previously described as one of the mechanisms of internalization for other types of QDs [22, 23]. Furthermore, upon 6 h incubation, both YbQDs and TbQDs accumulated in cytoplasm, but not in nucleus, as shown in Fig. 3d. This phenomenon was not identified for GdQDs, which were most likely quenched in culture medium and intracellular environment. This is in line with findings presented in Fig. 2a that demonstrate only negligible emission yields upon incubation in the NIE solution.

### Examination of Ln-doped QDs antimigratory properties and induction of ROS formation

**Fig. 4:**
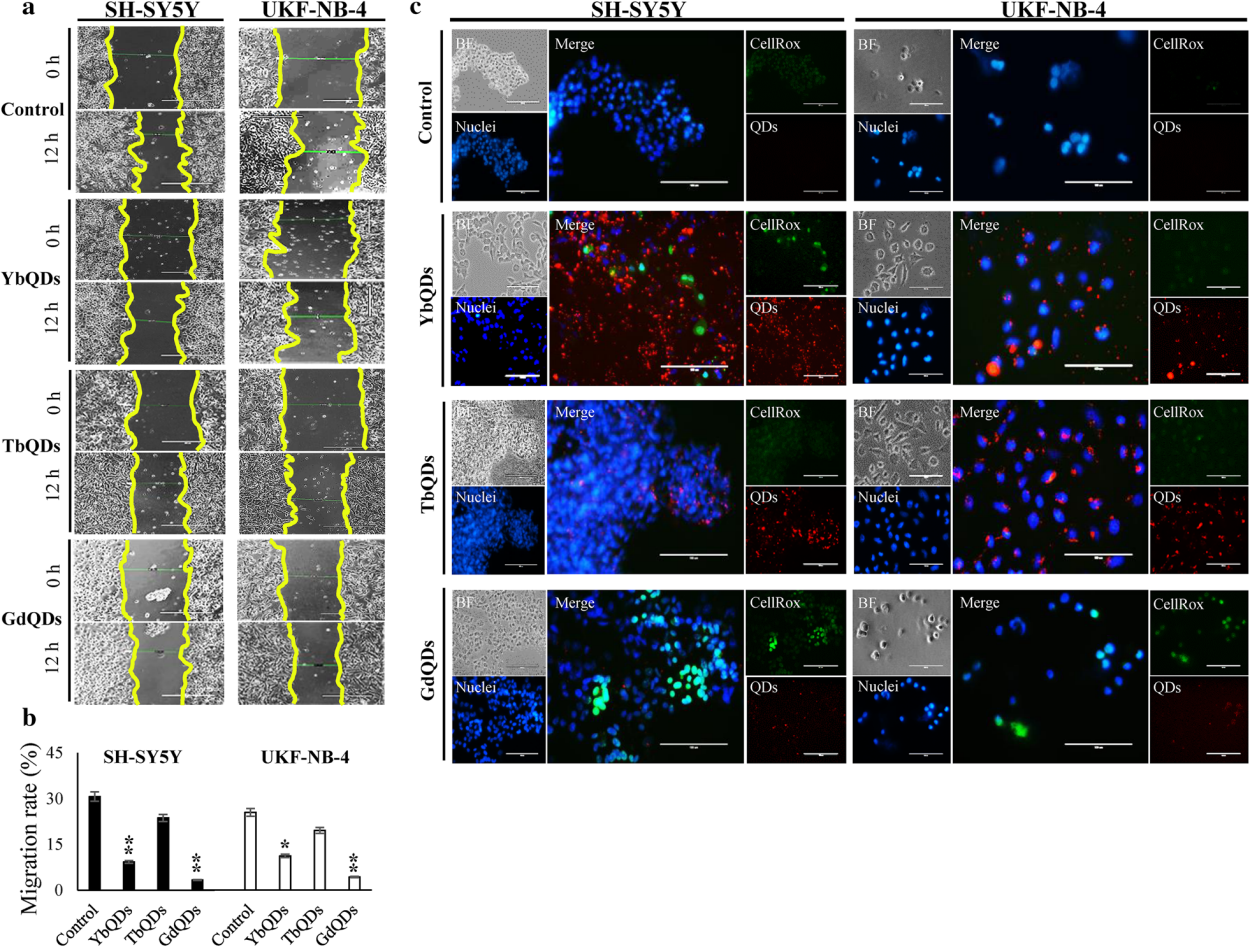
**a** Representative ambient photographs showing the effects of Ln-doped QDs on a migration of neuroblastoma cells. Photographs illustrate artificial wound at the experimental start-point (0 h) and migration of the cells after 12 h. Scale bar, 400 μm. **b** Bar graph demonstrating percentual migration rates. The values are expressed as the mean of six independent replicates (*n* = 6). Vertical bars indicate standard error. *p < 0.05, **p < 0.01 related to control non-treated cells. **c** Living cells fluorescence microscopy illustrating ROS (CellRox, green) formation in cells exposed to Ln-doped QDs (2 μM QDs for 3 h). Nuclei were counterstained with Hoechst 33342. Scale bars, 100 μm. *BF* bright field image

To further understand cytocompatibility of Ln-doped QDs, we analyzed collective sheet migration of both tested neuroblastoma cell lines after forming an artificial wound (Fig. 4a). As clearly seen from control analyses, both SH-SY5Y and UKF-NB-4 were slowly migrating cell lines, which is in a good agreement with van Nes and coworkers [24]. Figure 4b illustrates that the highest antimigratory activity was found for GdQDs (*p* < 0.01 for both cell lines). Contrary to that, TbQDs demonstrated only mild antimigratory activity. As among the most severe toxic effects of Cd-based nanomaterials is oxidative stress [25], we investigated whether cells exposed to Ln-doped QDs were attacked by ROS formation. Noteworthy, we revealed that the highest accumulation of intracellular ROS was caused by GdQDs exposure (Fig. 4c), while only negligible ROS were formed due to Tb- and YbQDs presence.

### Evaluation of biocompatibility of Ln-doped QDs and their effects on expression of selected proteins

**Fig. 5:**
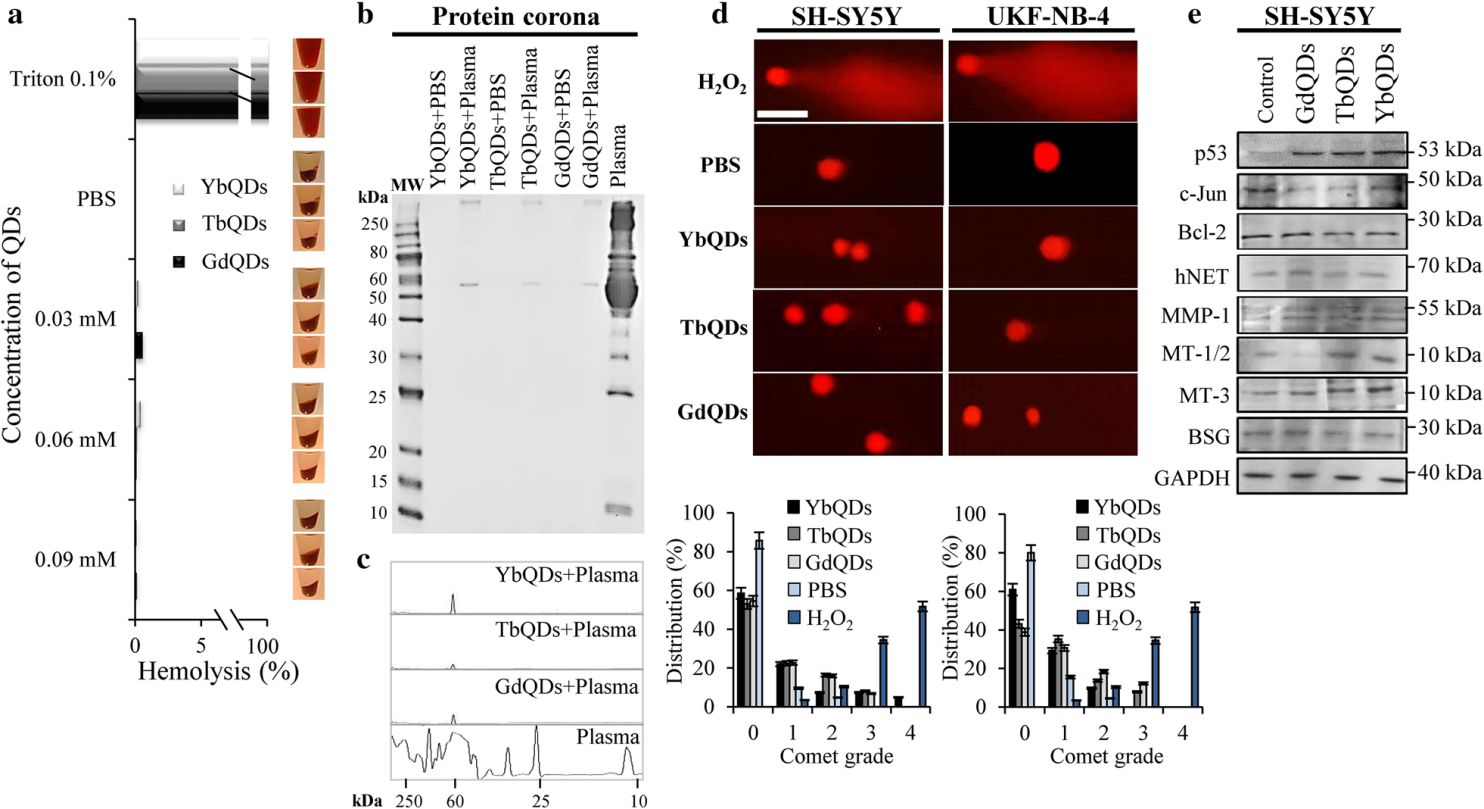
**a** Hemocompatibility of Ln-doped QDs assayed on human RBCs. PBS (pH 7.4) and 0.1% Triton X-100 were utilized as negative and positive controls, respectively. Images on the right side show representative photographs of exposed samples. The values are expressed as the mean of three independent replicates (*n* = 3). **b** Protein corona profiles obtained after 30 min incubation of Ln-doped QDs, with human plasma followed by extensive washing, elution and loading onto 12% SDS-PAGE. *MW* weight marker. **c** Proteins were further quantified by densitometric analysis. **d** Representative SCGE fluorescence micrographs showing negligible genotoxicity induced by Ln-doped QDs (2 μM, 24 h) in neuroblastoma cells, negative control [PBS (pH 7.4)], positive control (150 µM H_2_O_2_). Bar graphs below depict quantitation of index of damage (comet grades). Scale bar, 50 μm. The values are expressed as the mean of three independent replicates (*n* = 3). Vertical bars indicate standard error. **e** Immunoblots of whole-cell lysates showing regulatory effects of Ln-doped QDs on expression of selected proteins

First, we focused on interactions between Ln-doped QDs and human red blood cells (RBCs). As shown in Fig. 5a, all Ln-doped QDs demonstrated exceptional hemocompatibility, displaying no hemolysis compared to positive control (0.1% Triton X-100). Next, we focused on a formation of protein coronas, which are formed in plasma environment due to adsorption of plasma proteins on the surface of nanoparticles and negatively influence their behavior. Profiles of eluted proteins (Fig. 5b) revealed that only low degree of protein coronas was formed and Ln-doped QDs obviate most of unwanted interactions with plasma proteins. From densitometry, it can be concluded that proteins adsorbed on Ln-doped QDs are albumins (with approx. 66 kDa, Fig. 5c). Another pivotal aspect of nanoparticles’ biocompatibility is their genotoxicity. Hence, we performed single-cell gel electrophoresis (SCGE), which revealed that only low DNA fragmentation was present (primarily grades 1 and 2, Fig. 5d). Overall, we demonstrate that Ln-doped QDs possess exceptional biocompatibility, and could be potential candidates for in vivo applications. However, to fully prove this, a number of additional experiments will follow.

Finally, we attempted to identify a plausible role of Ln-doped QDs in regulating expression of proteins involved in fundamental biological processes. Figure 5e illustrates that all types of Ln-doped QDs stimulated expression of tumor suppressor p53 and inhibited expression of proto-oncogene protein c-Jun. Interestingly, TbQDs and YbQDs also stimulated expression of metallothioneins (MT-1/2 and MT-3), which are involved in metal homeostasis and subtly down-regulated anti-apoptotic protein Bcl-2.

### Designing immuno-labeling probes through site-directed conjugation of anti-hNET antibodies on Ln-doped QDs surface

**Fig. 6:**
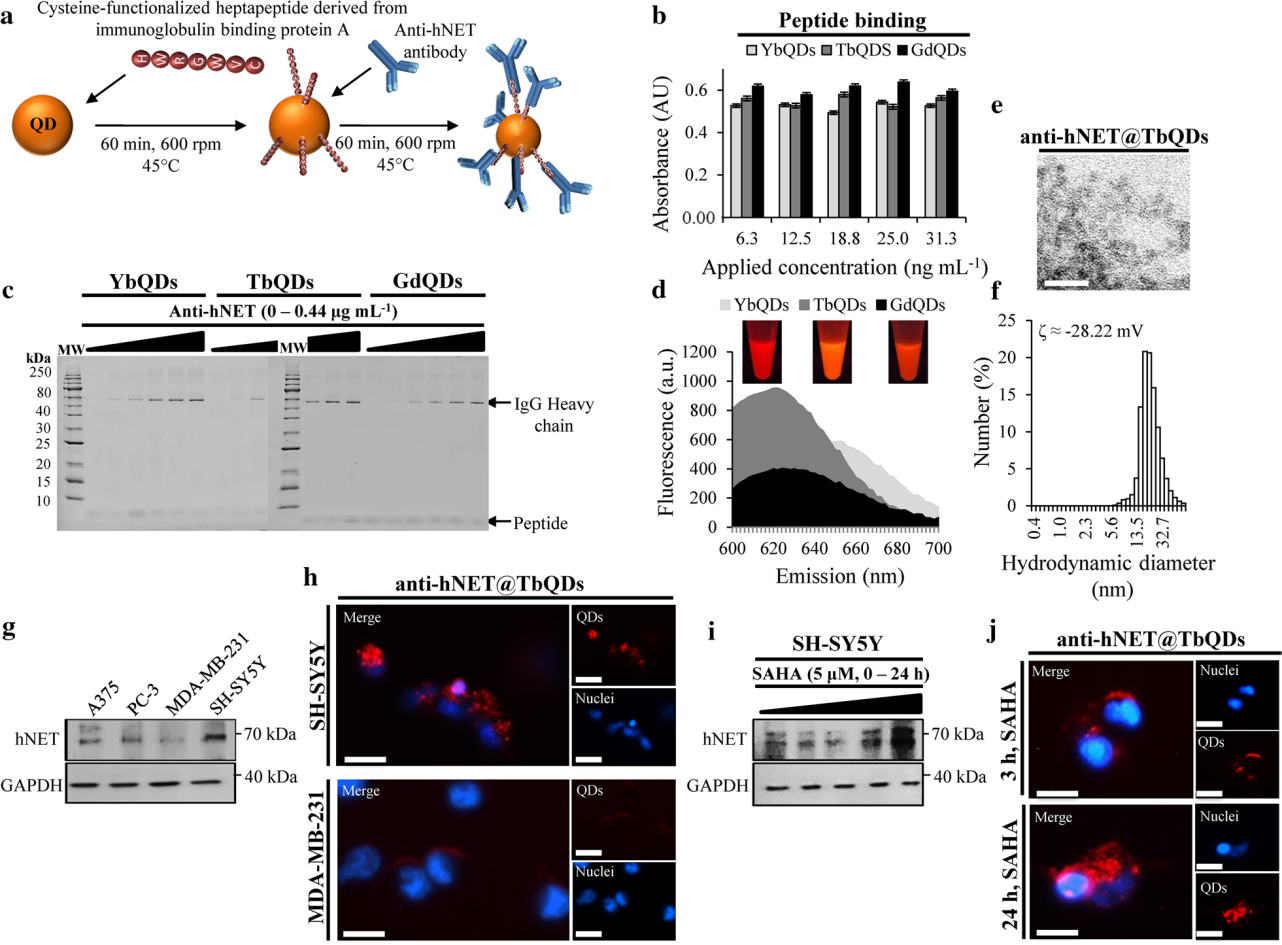
**a** Schematic depiction of a facile preparation of hNET-targeting Ln-doped QDs using cysteine-functionalized heptapeptide derived from immunoglobulin binding protein A for site-directed conjugation of anti-hNET antibodies. **b** Recovery of peptide linker binding onto surface of Ln-doped QDs. The values are expressed as the mean of three independent replicates (*n* = 3). Vertical bars indicate standard error. **c** SDS-PAGE showing components eluted from Ln-doped QDs. MW, weight marker. **d** Emission spectra of prepared constructs using λ_exc_ = 360 nm. Inserted are photographs of constructs after exposure to UV transillumination (λ = 312 nm). **e** TEM micrograph of anti-hNET@TbQDs. Scale bar, 100 nm. **f** Size distribution histogram of anti-hNET@TbQDs analysed by quasielastic DLS. Inserted is ζ-potential value analyzed in RS 100-fold diluted with Milli-Q water (pH 7.4) by Doppler microelectrophoresis. **g** Immunoblots of whole-cell lysates showing expression of hNET in four distinct cell lines. **h** Living cells fluorescence microscopy illustrating specific accumulation of anti-hNET@TbQDs (1 μM QDs upon 24 h incubation) on a surface of SH-SY5Y cells. Nuclei were counterstained with Hoechst 33342. Scale bars, 20 μm. **i** Immunoblots of whole-cell lysates showing stimulation of hNET in SH-SY5Y by SAHA. **j** Living cells fluorescence microscopy illustrating anti-hNET@TbQDs accumulation associated with SAHA-stimulated hNET expression. Nuclei were counterstained with Hoechst 33342. Scale bars, 20 μm

As a validation of applicability of Ln-doped QDs for in vitro visualization of receptor status, we performed a site-directed conjugation of anti-hNET antibodies onto Ln-doped QDs through synthetic peptide linker (Fig. 6a). The facile construction process benefits from a natural affinity of synthetic heptapeptide derived from protein A to Fc region of immunoglobulins. This enables site-directed conjugation of antibodies, which face their paratopes outward from the nanoparticles. To permit the peptide binding to Ln-doped QDs, *C*-terminus of peptide was functionalized with cysteine, having high affinity to metallic residues on the surface of QDs. All Ln-doped QDs had sufficient capacity to conjugate peptide linker and a plateau was reached using 6.3 ng/mL of peptide (Fig. 6b). Next, we verified a successful dose-dependent conjugation of anti-hNET antibodies onto Ln-doped QDs bearing peptide linker (Fig. 6c). The resulting immuno-probes retained their fluorescence, displaying emission maxima slightly shifted to 620–650 nm (Fig. 6d). Considering the best cytocompatibility attributes and the highest emission yield, we further focused on a characterization and testing of TbQDs (anti-hNET@TbQDs). Both TEM (Fig. 6e) and HDD shown in Fig. 6f confirmed a pronounced increase in size of anti-hNET@TbQDs (~ 20.8 nm) compared to un-modified TbQDs (~ 3.9 nm) that could presumably be explained by a mild aggregation due to the functionalization process.

### Immuno-labeling of hNET using anti-hNET@TbQDs

To investigate immuno-labeling performance of anti-hNET@TbQDs, we stratified four distinct cell lines using their hNET expression. The hNET is a clinically relevant target over-expressed in neuroendocrine tumors like neuroblastoma. As expected, the highest expression was identified for neuroblastoma cells (SH-SY5Y), while the lowest for breast cancer cells (MDA-MB-231, Fig. 6g). These two cell lines were consequently exposed to anti-hNET@TbQDs for 24 h at 37 °C and Fig. 6h illustrates considerable differences in accumulation of anti-hNET@TbQDs. To confirm this finding, we employed SAHA, which is known to cause an increase in hNET expression. Figure 6i demonstrates a time-dependent increase in hNET expression in SH-SY5Y cells pre-incubated with 5 μM SAHA. As shown in Fig. 6j, accumulation of anti-hNET@TbQDs correlated with SAHA expression. Importantly, even after 24 h incubation of cells with anti-hNET@TbQDs, we did not notice any morphological features related to apoptosis (chromatin condensation, apoptotic bodies or membrane blebbing). These data successfully confirmed applicability of anti-hNET@TbQDs as stable, cytocompatible immuno-probes for a facile visualization of hNET status.

## Discussion

In the present study, we investigated the cytocompatibility of three types of water-soluble Ln-doped QDs. We tested the following important hypotheses: (i) the surface doping of CdTe QDs could improve the cytocompatibility of QDs, (ii) emission yield of Ln-doped QDs is stable enough for cellular bio-labeling in vitro, and (iii) Ln-doped QDs could be efficient cytocompatible fluorophores for antibody-driven visualization of hNET status.

Ln-doped QDs were prepared by a facile two-step microwave-assisted method, which resulted in surface doping of CdTe QDs with Schiff base ([(2-[(E)-2-pyridylmethyleneamino]-*N*-[2-[(E)-2-pyridylmethylene-amino]ethyl]ethanamine)]. As evidenced by XPS, Schiff base was successfully utilized as a ligand for Gd^3+^, Tb^3+^ or Yb^3+^, respectively. Ln doping of nanoparticles became investigated many years ago, due to the unique spectroscopic signature of Ln^3+^ and their long fluorescence lifetime [15–17], which is fundamental for cellular imaging. Till now, several types of Ln-doped nanoparticles have been prepared and characterized [18–20]. However, to the best of our knowledge, there is a lack of data regarding biological behavior of Ln-doped QDs.

Our findings indicate that Ln-doped QDs were highly cytocompatible. On the other hand, un-doped CdTe QDs were toxic to the cells, which is in good agreement with previously published studies [7, 11, 26, 27]. It is a general fact, that cytocompatibility of QDs could be tuned by surface coating. Nevertheless, it has been shown that a longer exposure of surface-coated QDs to the bioenvironment can destabilize surface molecules, which can yield unprotected QDs to the cells [12]. Our cytotoxicity screenings demonstrated that toxic effects of Ln-doped QDs (particularly Yb- and TbQDs) appeared after exposure to very high concentrations of Ln-doped QDs, which, however, are significantly above the concentrations required for successful cellular imaging [8, 28]. One plausible explanation for Ln-doping-mediated improvement of CdTe QDs cytocompatibility is that Ln-doping causes a firm surface stabilization that inhibits the liberation of soluble, highly toxic Cd^2+^ ions from deteriorated QDs lattice as has previously been described by Derfus et al. [29].

Both, un-modified Yb- and TbQDs willingly internalized into cells and were distributed inside the cytoplasmic region, but not in the nuclei. In contrast, despite the fact that GdQDs were not found inside the cells by fluorescence microscopy, they demonstrated the highest cytotoxicity. It is a general fact that ROS formation plays a crucial role in the toxicological profile of nanomaterials [30]. Therefore, we further assessed Ln-doped QDs-induced formation of ROS. Interestingly, despite not being successfully tracked, presumably due to a quenching in culture medium and intracellular environment, GdQDs triggered pronounced formation of ROS compared to Tb- and YbQDs. This could be explained by a release of Cd^2+^ due to an imperfect or unstable doping, which also resulted in destabilization and quenching of GdQDs by substances contained in culturing media [31]. Moreover, ROS formation could also occur due to Gd presence, which has previously been described to induce both oxidative and endoplasmic reticulum stress in neurons [32]. However, to elucidate the distinctness in behavior of GdQDs, further experiments might follow.

Despite in vivo application of Ln-doped QDs being beyond the aim of this work, we also carried out testing of their biocompatibility. It has been found that several types of nanoparticles possess considerable hemotoxicity, which weakens their potential to be used in nanomedicine [33]. This is most likely linked with considerably negative ζ-potentials of Ln-doped QDs, which might prevent RBCs (having charge of − 15 mV [34]) from interacting even at relatively high concentrations of Ln-doped QDs. At the cellular level, Cd^2+^ alone or in Cd-based QDs could induce DNA strand breakage, nuclei damage and lipid peroxidation through ROS formation [35, 36]. Noteworthy, we show that only negligible DNA fragmentation was caused by exposure of neuroblastoma cells to Ln-doped QDs, which underpin an importance of Ln^3+^ as surface dopants. In addition, Ln-doped QDs exhibited only a minor formation of protein coronas. These are formed upon contact with bodily fluids when plasma proteins are adsorbed onto nanoparticles surface. This process can meticulously impair nanoparticle properties [37], and is thus one of the pivotal aspects for intravenous applications.

As QDs can have distinct regulatory activities [38, 39], we attempted to identify an influence of Ln-doped QDs on expression of proteins involved in various biological processes. Our findings show an increase in expression of p53 that is a key regulator of cell cycle. Similar results were achieved by Choi et al. [40], who suggested that p53 translocation contributes to QDs-induced toxicity. Contrary to that, we show that Ln-doped QDs can stimulate p53 without profound toxicity, which is most likely associated with only a subtle repression of anti-apoptotic protein Bcl-2. We also found Ln-doped QDs-mediated down-regulation of c-Jun and slight stimulation of metal homeostasis proteins MT-1/2 and MT-3. However, importance of these results needs to be confirmed by additional functional studies, which could disclose conceivable applicability of Ln-doped QDs in combination therapy of cancer diseases.

Finally, as a proof-of-concept, we designed a conjugation system relying on site-directed conjugation of antibody through synthetic peptide linker. Similar approach has already been employed in our previous studies for conjugation of antibodies onto different types of nanoparticles [41, 42]. We focused on hNET (also known as SLC6A2) that actively transports norepinephrine into adrenal chromaffin cells and pre-synaptic terminals by Uptake-1 [43]. It is worth noting that hNET is often over-expressed in neuroendocrine tumors and is targeted by radiolabeled norepinephrine analog mIBG (utilizing Uptake-1) [21]. Therefore, hNET expression is a prerequisite for a successful therapy. We present that Ln-doped QDs can be seamlessly conjugated for immuno-labeling. We also show that TbQDs act as stable, cytocompatible fluorophore (being efficiently localized after 24 h incubation in normal culture medium). This is advantageous to many organic fluorescent dyes that undergo oxidative photo bleaching, which tends to produce free radical breakdown products [44]. The presented approach is versatile and applicable for immuno-visualization of any membrane-bound protein. We also anticipate that Ln-doped QDs could be efficient fluorophores for immunocytochemistry in fixed and permeabilized cells; however, this needs experimental verification.

## Conclusion

In conclusion, we present facile two-step synthesis of cytocompatible Ln-doped QDs. Ln^3+^ are doped in a second step of synthesis through Schiff base. It was shown that Ln-dopants have considerable effects on cytotoxicity of bare CdTe QDs making them cytocompatible fluorophores with exceptional fluorescence stability. Despite in vivo application of Ln-doped QDs is beyond the scope of this study, we found that Ln-doped QDs possess remarkable biocompatibility, which is an important prerequisite for in vivo imaging fluorophores. This will be investigated in following studies focused on Ln-doped QDs tissue bioaccumulation, immunogenicity and a fluorescence penetration depth. Additionally, we carried out simple and versatile approach for a site-directed conjugation of antibodies. Whole conjugation process resulted in specific antibody-driven fluorophore with pronounced fluorescence stability. Overall, Ln-doping of QDs seems to be a way to improve their applicability in various bio-labeling experiments.

## Methods

### Chemicals

Listed chemicals were purchased from Sigma-Aldrich (St. Louis, MO, USA) in ACS purity, unless noted otherwise.

### Synthesis of Gd, Tb and Yb-Schiff base complexes

The Schiff base, [(2-[(E)-2-pyridylmethyleneamino]-*N*-[2-[(E)-2-pyridylmethylene-amino]ethyl]ethanamine)], was prepared according to [45] with slight modifications. Briefly, 1080 µL of diethylenetriamine and 1900 µL of 2-pyridinecarboxaldehyde were mixed and heated under reflux in methanol for 6 h. After cooling, methanol was added to make the final volume up to 50 mL to get the desired Schiff base solution. In separate beakers, methanol (10 mL) was mixed with aqueous solutions of gadolinium(III) nitrate or terbium(III) nitrate or ytterbium(III) nitrate and then the Schiff base solution (5 mL) was added to them subsequently. The solutions were mixed well at 40 °C for 2 h and the volume was made up to 100 mL with deionized water.

### Synthesis of CdTe QDs and their surface modification by Gd-, Tb- and Yb-Schiff base complexes

Microwave preparation of the CdTe QDs was carried out according to our previous study [46]. Briefly, 53.2 mg of cadmium acetate was mixed with 86 mL of ACS-grade water on a magnetic stirrer, followed by the addition of 60 mg of mercaptosuccinic acid. Next, 1.8 mL of an ammonia solution (1 M) was added to pH 7.0. Then, 1.5 mL of a sodium telluride solution (221 mg/mL in water, w/v) was added, and the solution was mixed well. Subsequently, 50 mg of sodium borohydride was added to the solution, which was stirred for approximately 2 h until bubble formation ceased, and the volume of the solution was brought to 100 mL with ACS water. 2 mL of this solution was removed; placed in a small glass vessel and heated at 300 W under microwave irradiation (Multiwave 3000, Anton-Paar GmbH, Graz, Austria). Next, the Gd or Tb or Yb-Schiff base complexes were added, followed by heating using 300 W under microwave irradiation to prepare the GdQDs, TbQDs and YbQDs respectively. The QDs were filtered through 0.22 µm membranes and subsequently dialyzed using cellulose acetate membrane (1 kDa pore size) in deionized water several times to remove the unreacted initiators. Then, the QDs were dispersed in deionized water for further use.

### Physico-chemical characterization

To evaluate their colloidal stability, Ln-doped QDs were dispersed in the RS (6.5 g sodium chloride, 0.42 g potassium chloride, 0.25 g calcium chloride and 0.2 g of sodium bicarbonate dissolved in 1 L of water, pH 7.4), placed in the stationary rack and kept at 25 °C. Photographic documentation of possible sedimentation was performed every 12 h. To investigate morphology of Ln-doped QDs, TEM Tecnai F20 TEM (FEI, Eindhoven, Netherlands) was used. ζ-Potential was evaluated using Doppler microelectrophoresis on Zetasizer Nano ZS90 (Malvern instruments, Malvern, UK) as well as particle HDD analysis by DLS. The refractive index (RI) of dispersive phase was 0.79 for YbQDs, 1.7 for TbQDs and 1.8 for GdQDs, respectively and the RI of dispersive environment was 1.333 for all tested samples. For each size measurement, Zen0040 disposable cuvettes (Brand GmbH, Wertheim, Germany) were used, containing 50 μL of sample. For each ζ-potential measurement, disposable cells DTS1070 (Brand GmbH) were employed, with number of runs varied between 20 and 40, and calculations considered the diminution of particle concentration based on the Smoluchowski model, with an F(ka) of 1.5. Analyses were performed in RS (100-fold diluted with Milli-Q water), which is an isotonic solution relative to the plasma. Prior to measurements, samples were incubated at 25 °C for 15 min. The fluorescence QY (%) of the Ln-doped QDs was determined using rhodamine 6G as a reference according to a reported protocol [47]. XPS analyses were carried out with Axis Ultra DLD spectrometer using a monochromatic Al Kα (hν = 1486.7 eV) X-ray source operating at 75 W (5 mA, 15 kV). The spectra were obtained using an analysis area of ~ 300 × 700 µm. The wide spectra were measured with the step size 0.7 eV and 160 eV pass energy. Spectra were analysed using CasaXPS software (version 2.3.15) and have been charge corrected to the main line of the carbon C 1 s spectral component set to 284.8 eV. FT-IR spectra were collected using a Nicolet iS10 FT-IR spectrometer with diamond attenuated total reflectance attachment (Thermo Electron Inc., San Jose, USA). Spectra were recorded at 25 °C from 4000 to 650 cm^−1^ at a resolution of 2 cm^−1^. Each spectrum was acquired by merging 128 interferograms. For all applications, concentration of Ln-doped QDs was standardized to an equal cadmium content. Prior to analyses using atomic absorption spectrometer 280Z (Agilent Technologies, Santa Clara, CA, USA), samples were digested using nitric acid (65% *v/v*) and hydrogen peroxide (30% *v/v*) in Multiwave 3000 (Anton-Paar GmbH).

### Analyses of fluorescence stability and 2D emission–excitation spectra maps

Fluorescence stability and HDD of Ln-doped QDs was investigated in three solutions mimicking distinct physiological environments: (i) RS mimicking plasma environment (composition described above), (ii) solution mimicking endosomal environment (0.142 g disodium phosphate, 6.650 g sodium chloride, 0.071 g sodium sulfate, 0.029 g calcium chloride dihydrate, 0.45 g glycine and 4.1 g potassium hydrogen phthalate in 1 L of water, pH 5.0) and (iii) solution mimicking neutral intracellular fluid (0.212 g magnesium chloride hexahydrate, 6.415 g sodium chloride, 0.318 g calcium chloride tetrahydrate, 0.179 g sodium sulfate decahydrate, 0.148 g disodium phosphate, 2.703 g sodium bicarbonate, 0.18 g sodium tartrate dihydrate, 0.144 g trisodium citrate dihydrate, 0.175 g sodium lactate, 0.118 g glycine and 0.172 g sodium pyruvate in 1 L of water, pH 6.9). The 2D fluorescence emission–excitation spectral maps of Ln-doped QDs were analysed using the Tecan Infinite 200 PRO (Tecan, Maennedorf, Switzerland). The 2D fluorescence datasets were obtained in form of a triangular matrix with excitation wavelengths of 230–850 nm with 5 nm step and scan emission range (excitation wavelength + 35)–850 nm with 5 nm step. Shorter wavelengths were set to zero. All measurements were performed at 30 °C.

### Cell lines and culture conditions

Cell lines used in this study were: (i) the UKF-NB-4 cell line that was established from recurrent bone marrow metastasis of high-risk neuroblastoma, (ii) the SH-SY5Y human cell line established from a bone marrow metastasis of a 4-years-old female neuroblastoma, (iii) the MDA-MB-231 human cell line established from a pleural effusion of a 51-year-old woman with metastatic breast cancer, (iv) the A375 human cell line derived from a 54-years-old female with malignant melanoma and (v) the PC-3 human cell line established from bone metastasis of grade IV of prostate cancer in a 62-year-old Caucasian male. Except for UKF-NB-4 cell line that was a kind gift from prof. Tomas Eckschlager (Department of Pediatric Hematology and Oncology, University Hospital Motol, Prague, Czech Republic), cell lines were purchased from Health Protection Agency Culture Collections (Salisbury, UK). UKF-NB-4 were cultured in IMDM. The rest of cell lines were cultured in RPMI-1640. Media were supplemented with 10% foetal bovine serum, with penicillin (100 U/mL) and streptomycin (0.1 mg/mL). The cells were maintained at 37 °C in a humidified incubator Galaxy^®^ 170 R (Eppendorf, Hamburg, Germany).

### Testing the effects of QDs on cellular proliferation

The viability was assayed using MTT (3-(4,5-dimethylthiazol-2-yl)-2,5-diphenyltetrazolium bromide) assay. Briefly, the suspension of 5000 cells in 50 µL medium was added to each well of microtiter plates, followed by incubation for 24 h at 37 °C with 5% CO_2_ to ensure cell growth. To determine the effects on cellular proliferation, YbQDs, TbQDs, GdQDs and CdTe QDs (0.5–0.001 mM) were applied. Treatment was carried out for 24 h. Then, 10 µL of MTT [5 mg/mL in phosphate buffered saline (PBS)] was added to the cells and the mixture was incubated for 4 h at 37 °C. After that, MTT-containing medium was replaced by 100 µL of 99.9% dimethyl sulfoxide and, after 5 min incubation, absorbance of the samples was determined at 570 nm using Infinite 200 PRO (Tecan).

### Investigation of QDs-induced endocytosis

After 6 h of treatment with 2 μM QDs, living cells were stained using the pHrodo™ Green Dextran, 10000 MW (Thermo Fisher Scientific, Waltham, MA, USA) according to the manufacturer’s protocol. Nuclei were counterstained with Hoechst 33342. Then, cells were visualized using the EVOS FL Auto Cell Imaging System (Thermo Fisher Scientific, Waltham, MA, USA).

### Wound-healing assay (Scratch test)

The cells were pipetted into 6-well plate to reach the confluence of ~ 100%. After seeding of cells on the bottom of a plate, a pin was used to scratch and remove cells from a discrete area of the confluent monolayer to form a cell-free zone. After that, cells were re-suspended in a fresh medium enriched with 2 μM QDs. After 12 h, the pictures of cells were taken and compared with pictures obtained in 0 h, using TScratch software (CSElab, Zurich, Switzerland).

### Fluorescence microscopy of ROS

Cells were cultivated directly on microscope glass slides (75 × 25 mm, thickness 1 mm, Fischer Scientific, Czech Republic) in Petri dishes. After treatment (2 μM QDs, 3 h), microscope glass slides with a monolayer of cells were removed from Petri dishes, rinsed with cultivation and directly used for analysis of ROS using CellROX^®^ Green Reagent (Thermo Fisher Scientific) according to manufacturer’s instructions. For nuclei counterstaining, Hoechst 33342 was employed. Cells were visualized using the EVOS FL Auto Cell Imaging System (Thermo Fisher Scientific).

### Hemocompatibility

Hemocompatibility of Ln-doped QDs was assayed using human RBCs. Fresh blood sample was withdrawn aseptically by antecubital venipuncture of healthy human donor with signed informed consent. Then, RBCs were obtained according to Evans et al. [48]. RBCs suspensions were washed with 150 mM NaCl solution three-to-five times. Then, different concentrations of Ln-doped QDs (0.03–0.09 mM), diluted in PBS were mixed with RBCs and incubated for 1 h at 37 °C. The degree of hemolysis was determined by measuring the absorbance of the supernatant at 540 nm, after centrifugation and calculated according to the following equation: %*hemolysis* = [(*A*_*t*_ − *A*_*c*_)/*A*_100%_ − *A*_*c*_)] × 100, where *A*_*t*_ is the absorbance of the supernatant from samples incubated with the QDs; *A*_*c*_ is the absorbance of the supernatant from negative control (PBS, pH 7.4) and; *A*_*100%*_ is the absorbance of the supernatant from positive control (0.1% Triton X-100), which causes complete lysis of RBCs.

### Analysis of protein corona formation around Ln-doped QDs

Immediately after blood collection, plasma was isolated from whole blood by centrifugation (3000×*g*, 5 min). Subsequently, QDs (2 μM in PBS) were incubated in plasma at 1:1 ratio (v/v) in order to mimic the protein concentration in vivo (50% plasma in blood). The incubation was done for 35 min at 37 °C under continuous agitation. The protein coronas were recovered after 10 min centrifugation at 15,000×*g* and washed three times with cold PBS to remove unbound proteins. Finally, the proteins were eluted by adding sodium dodecyl sulfate (SDS), separated by 12.5% sodium dodecyl sulfate polyacrylamide gel electrophoresis (SDS-PAGE) and stained by Coomassie brilliant blue (CBB). Gels were visualized using Azure c600 (Azure Biosystems, Dublin, CA, USA). Plasma proteins were quantified by densitometric analysis with the AzureSpot software (Azure Biosystems).

### SCGE for analysis of DNA fragmentation

The cells were plated at a density of 10^6^ cells/well in six-well dishes and treated with QDs (2 μM) for 24 h. As a control, 150 µM H_2_O_2_ was employed. After harvesting, about 15 μL of the cell suspension was mixed with 75 μL of 0.5% low melting point agarose (CLP, San Diego, CA, USA) and layered on one end of a frosted plain glass slide. Then, it was covered with a layer of the low melting agarose (100 μL). After solidification of the gel, the slides were immersed in a lysing solution (2.5 M NaCl, 100 mM Na_2_EDTA, 10 mM Tris, pH 10) containing 1% Triton X-100 and 10% DMSO), with an overnight incubation at 4 °C. A cold alkaline electrophoresis buffer was poured into the chamber and incubated for 20 min at 4 °C. The electrophoresis was carried at 4 °C, (1.25 V/cm, 300 mA) for 30 min. The slides were neutralized (0.4 M Tris, pH 7.5) and then stained with ethidium bromide (EtBr, 2 µg/mL). The cells were analysed under fluorescence microscope EVOS FL Auto Cell Imaging System (Thermo Fisher Scientific) and classified according to the shape of the fluorescence of the comet tail [0 (*no visible tail*) to 4 (*significant DNA in tail*)].

### Western blotting

Total cellular proteins were extracted with 100 µL of RIPA buffer containing protease inhibitor cocktail. After electrophoresis, the proteins were electro transferred onto the Immuno-Blot^®^ PVDF membrane (Bio-Rad, Hercules, CA, USA) and a non-specific binding was blocked with 10% (w/v) non-fat fresh milk for 1 h at 37 °C. Membranes were incubated with primary mouse anti-p53 (dilution 1:250), mouse anti-c-Jun (1:250), mouse anti-Bcl-2 (1:200), mouse anti-hNET (1:200), mouse anti-MMP-1 (1:200), mouse anti-MT-3 (1:200), goat anti-EMMPRIN (1:200), mouse anti-MT1 + 2 (1:200) and mouse anti-GAPDH (1:700) overnight at 4 °C. After washing, membranes were incubated with relevant horseradish peroxidase-labeled secondary antibodies (1:5000, Dako, Santa Clara, CA, USA) for 1 h at 20 °C. Signals were developed using Clarity Western ECL Blotting Substrate (Bio-Rad) and blots were visualized using Azure c600 imager (Azure Biosystems).

### Site-directed functionalization of QDs using anti-hNET antibodies

For site-directed conjugation of antibodies, HWR heptapeptide (HWRGWVC, 943.0912 Da), derived from immunoglobulin-binding protein A, was prepared on Liberty Blue Peptide Synthesizer (CEM, Matthews, NC, USA) by Fmoc solid-phase synthesis. Purity and mass distribution of crude peptide was analysed using high-performance liquid chromatography with UV detection (ESA Inc., Chelmsford, MA, USA) and matrix-assisted laser ionization/desorption time-of-flight mass spectrometry (Bruker ultrafleXtreme, Bruker Daltonik GmbH, Germany). For cysteine-driven conjugation of peptide onto QDs surface, equal volumes of QDs (2 µM) and HWR peptide (6.25; 12.5; 18.75; 25.00, and 31.25 ng/mL) were mixed for 60 min at 2000×*g* and 45 °C. Then, the solution was filtered through Amicon^®^ Ultra-3K and conjugated peptides were quantified by absorbance at 280 nm on Tecan Infinite 200 PRO (Tecan). Further, anti-hNET antibodies were conjugated (0.09–0.43 μg/mL) by mixing (60 min, at 2000×*g* and 37 °C). To investigate the conjugation recovery, the antibodies were eluted by adding SDS, separated by 12% SDS-PAGE and stained by CBB. Gels were visualized using Azure c600 (Azure Biosystems). The size and ζ-potential of whole construct were analysed using TEM (Tecnai F20 TEM, FEI) and DLS with Doppler microelectrophoresis (Zetasizer Nano ZS90, Malvern instruments). Prior to measurements, samples were incubated in RS at 25 °C for 15 min.

### Visualization of hNET expression and stimulation of hNET expression using SAHA

In all experiments, the binding efficiency of antibodies conjugated to TbQDs (hereinafter referred to as anti-hNET@TbQDs) was investigated upon 24 h incubation with cells at 37 °C and visualized using the EVOS FL Auto Cell Imaging System (Thermo Fisher Scientific). To verify specificity of anti-hNET@TbQDs towards hNET, we employed suberanilohydroxamic acid (SAHA or vorinostat) that causes a dose-dependent increase in expression of hNET. SH-SY5Y cells were treated with 5 μM SAHA (0–24 h) and the expression was validated on Western blots following protocol described above.

### Descriptive statistics

For the statistical evaluation of the results, the mean was taken as the measurement of the main tendency, while standard deviation was taken as the dispersion measurement. Differences between groups were analysed using paired *t* test and ANOVA. For analyses Software Statistica 12 (StatSoft, Tulsa, OK, USA) was employed.

## Abbreviations

QDs: quantum dots
Ln: lanthanides
hNET: human norepinephrine transporter
QY: quantum yield
ROS: reactive oxygen species
RS: Ringer’s solution
DLS: dynamic light scattering
HDD: hydrodynamic diameter
TEM: transmission electron microscopy
XPS: X-ray photoelectron spectroscopy
FT-IR: Fourier transform infrared spectroscopy
EE: endosomal environment
NIE: neutral intracellular environment
BF: bright field image
RBCs: red blood cells
SCGE: single-cell gel electrophoresis
SDS-PAGE: sodium dodecyl sulfate polyacrylamide gel electrophoresis
PBS: phosphate buffered saline
mIBG: metaiodobenzylguanidine
MTT: 3-(4,5-dimethylthiazol-2-yl)-2,5-diphenyltetrazolium bromide
